# Renal Type A Intercalated Cells Contain Albumin in Organelles with Aldosterone-Regulated Abundance

**DOI:** 10.1371/journal.pone.0124902

**Published:** 2015-04-13

**Authors:** Thomas Buus Jensen, Muhammad Umar Cheema, Agata Szymiczek, Helle Hasager Damkier, Jeppe Praetorius

**Affiliations:** Department of Biomedicine, Health, Aarhus University, Aarhus, Denmark; Anatomy, SWITZERLAND

## Abstract

Albumin has been identified in preparations of renal distal tubules and collecting ducts by mass spectrometry. This study aimed to establish whether albumin was a contaminant in those studies or actually present in the tubular cells, and if so, identify the albumin containing cells and commence exploration of the origin of the intracellular albumin. In addition to the expected proximal tubular albumin immunoreactivity, albumin was localized to mouse renal type-A intercalated cells and cells in the interstitium by three anti-albumin antibodies. Albumin did not colocalize with markers for early endosomes (EEA1), late endosomes/lysosomes (cathepsin D) or recycling endosomes (Rab11). Immuno-gold electron microscopy confirmed the presence of albumin-containing large spherical membrane associated bodies in the basal parts of intercalated cells. Message for albumin was detected in mouse renal cortex as well as in a wide variety of other tissues by RT-PCR, but was absent from isolated connecting tubules and cortical collecting ducts. Wild type I MDCK cells showed robust uptake of fluorescein-albumin from the basolateral side but not from the apical side when grown on permeable support. Only a subset of cells with low peanut agglutinin binding took up albumin. Albumin-aldosterone conjugates were also internalized from the basolateral side by MDCK cells. Aldosterone administration for 24 and 48 hours decreased albumin abundance in connecting tubules and cortical collecting ducts from mouse kidneys. We suggest that albumin is produced within the renal interstitium and taken up from the basolateral side by type-A intercalated cells by clathrin and dynamin independent pathways and speculate that the protein might act as a carrier of less water-soluble substances across the renal interstitium from the capillaries to the tubular cells.

## Introduction

Albumin is a major plasma protein responsible for the oncotic pressure of the blood [1] and a carrier of substances such as free fatty acids, steroid hormones, bilirubin, and Ca^2+^ [2]. Serum albumin is produced by the hepatocytes and is mainly kept within the blood stream after hepatic exocytosis. The fraction of albumin filtered by the kidney is quite modest because of its negative charge, globular shape and molecular weight (66.5 kDa) [3]. Filtered albumin is normally almost completely reabsorbed (>99%) by receptor mediated endocytosis in proximal tubules [4–7] leaving urine practically albumin free.

Recent studies have detected albumin in late distal convoluted tubules (late DCT), connecting tubules (CNT) and cortical collecting ducts (CCD) by mass spectrometry [8,9]. Although albumin might be a contaminant it remains possible that albumin is either taken up by cells in the late DCTs, CNTs and CCDs or synthesized in these epithelial cells. The first option would suggest that these tubular segments endocytose any remaining filtered albumin or they may take up albumin from the interstitium. The late DCT, CNT and CCD contain several different cell types. The intercalated cells play a critical role in acid/base balance [10] and principal cells of the CCD govern the fine-tuning of Na^+^ reabsorption, K^+^ secretion and total body fluid volume [11]. Aldosterone produced in the cortex of the adrenal gland, is intricately involved in the regulation of ion transport by all of these cell types [12–14]. In the blood, aldosterone is partially bound to albumin and the free fraction of the hormone determines the effect on the target cells, as for other protein-bound hormones. In a previous study, quantitative mass spectrometry suggested that 24-hours aldosterone administration decreased albumin abundance in the late DCT, CNT and CCD [8]. The cellular identity of the putatively albumin containing cells remains elusive, as mass spectrometry detected albumin in studies of both isolated intercalated cells [9] and non-intercalated late DCT, CNT and CCD cells [8].

Validation of and extending on these observations would potentially be of great physiological and even clinical importance and to spur further investigations into the putative significance of distal tubular uptake of urinary or even interstitial albumin. Thus, we undertook the current study 1) to establish whether albumin is present in late DCT, CNT and CCD collecting duct cells, and if so 2) to identify the albumin containing cell type and intracellular localization of albumin, 3) to suggest the source of albumin for tubular uptake, and 4) to validate the effect of aldosterone on tubular albumin contents.

## Methods

### Animals

A total of 18 wild-type male c57bl/6 mice (Taconic) were divided into three groups for 48-hour experiments with injections of vehicle (sunflower seed oil) or 2.0 mg/kg aldosterone in vehicle [8]. Controls received 2 vehicle injections at 0 and 24 hours; the 24-hour aldosterone group received vehicle at 0 hours and aldosterone after 24 hours, while the 48-hr aldosterone group received aldosterone injections at both 0 and 24 hours. Transgenic mice expressing eGFP driven by the TRPv5 promoter were used for isolating connecting tubules and cortical collecting ducts for RT-PCR [15]. The authors are licensed to breed these GMO mice and the animal experiments were performed according to a license issued by the Animal Experiments Inspectorate, Ministry of Food, Agriculture and Fisheries—Danish Veterinary and Food Administration.

### Tissue fixation and immunohistochemical staining

Kidneys were perfused with 4% paraformaldehyde in PBS, post-fixed for 30 minutes and dehydrated in graded ethanol (70%, 96%, and 99%) for 2 hours each and left overnight in xylene. The tissue was embedded in paraffin wax, cut into 2 μm thick sections on a rotary microtome (Leica), and placed on Super Frost slides. Sections were dewaxed in xylene and rehydrated in graded ethanol. Endogenous peroxidase was blocked after 96% ethanol in 35% H_2_O_2_ in methanol. To retrieve antigens, sections were boiled in a microwave oven in TEG-buffer, pH 9, with 10 mM Tris and 0.5 mM EGTA. Aldehydes were quenched in 50 mM NH_4_Cl in PBS, and the sections were blocked in 0.1% skim milk powder, 0.2% gelatin, 0.05% Saponin in PBS. Finally, sections were incubated with primary antibody in 0.1% skim milk powder, 0.3% Triton X-100 in PBS overnight at 4°C, and rinsed in 0.1% skim milk powder, 0.2% gelatin, and 0.05% Saponin in PBS.

For light microscopy, sections were incubated 1 hour with horseradish peroxidase conjugated secondary antibody in 0.1% skim milk powder, 0.3% Triton X 100 in PBS and washed in 0.1% skim milk powder, 0.2% gelatin, and 0.05% saponin in PBS before visualization with diaminobenzidine in 35% H_2_O_2_ for 10 minutes. Finally, the sections were counterstained with Mayers hematoxylin and rinsed in running tap water before dehydration in graded ethanol and xylene and mounting with coverslips using Eukitt (CellPath). For immunofluorescence staining, the blocking of peroxidase was omitted and fluorophore tagged secondary antibodies applied. Coverslips were mounted with a hydrophilic mounting medium containing antifading reagent (DAKO, glycergel).

### Immuno-gold electron microscopy

Small renal cortical tissue blocks were cut from the fixed mouse kidneys, infiltrated overnight in 0.01M PBS with 2.3 M sucrose and 2% paraformaldehyde, mounted on holders, and rapidly frozen in liquid nitrogen. Tissue blocks with random orientation were cryosectioned with a Reichard FCS Reichert Ultracut S (Leica Microsystems, Wetzlar, Germany) at -120°C. The 80-nm cryosections were first blocked by incubation in PBS containing 0.05 M glycine and 0.1% skim milk powder. The sections were then incubated for 1 hour at room temperature with antibody against albumin in PBS containing 0.1% skim milk powder. The primary antibody was visualized using 10 nm gold-conjugated secondary antibodies in PBS with 0.1% skim milk powder and polyethyleneglycol (5 mg/ml). The cryosections were stained 10 minutes with 0.3% uranyl acetate in 1.8% methyl-cellulose and examined in a FEI Morgagni electron microscope.

### Cell culture and albumin uptake studies

Low-resistance MDCK cells at passage 62 to 72 (American Type Culture Collection, Rockville, MD) were grown in Dulbecco’s modified Eagle’s medium. The medium was supplemented with 10% fetal bovine serum (Gibco, Grand Island, NY) and 2 mM glutamine. Cells were grown to confluency at 37°C in 5% CO_2_/95% atmospheric air on 6-well permeable polyester support with 0.4 μm poresize (Transwell, Costar). Fluorescein conjugated albumin (Sigma) was added to obtain 62.5 μg/ml as final concentration for 8 or 24 hours. Clathrin mediated endocytosis was inhibited by 10 μg/ml Chlorpromazine (Sigma) and dynamin dependent endocytosis by 80 μM Dynasore (Sigma D7693). Peanut agglutinin rhodamine 1:100 (Vector Laboratories) was added to the top bath medium and excess lectin was removed by rinsing once in PBS. Cells were fixed for 10 minutes in 4% PFA and rinsed twice in PBS.

### Image acquisition and processing

Brightfield imaging was performed on a Leica DMRE light microscope with PC APO 63x/1.32–0.6 NA and PC FLUOTAR 25x/0.75 NA oil immersion objectives, and a Leica DC 300 digital camera. Fluorescence imaging was performed on a Leica DM IRE2 inverted confocal microscope using a Leica TCS SP2 laser module and an HCX PC APO CS 63x/1.32 NA oil objective. Images were acquired using the following settings: 8 bit image depth, 1024x1024 pixel resolution, and an image averaging of 6 frames. Laser power and settings for PMT gain and offset were kept constant for each antibody and adjusted to the brightest section. Image-Pro Analyzer (Media Cybernetics) and ImageJ (NIH) were used for semi-quantitation and merging the confocal images as well as stacking. For semi-quantitation, the tubular cell area and the background-corrected fluorescence signal were determined [8]. The fluorescence signal was then normalized to the total tubule cell area and averaged for four images from each mouse kidney. Image stacks were also processed by Imaris software (Bitplane) for top view and oblique displays.

### Isolation of mRNA and reverse transcription—polymerase chain reaction (RT-PCR)

EGFP expressing tubules were isolated according to the SuperScript III CellsDirect cDNA Synthesis System (Invitrogen) from male TRPv5-eGFP expressing mice [15]. The mRNA from renal cortex homogenate as well as other tissues was extracted in TRI Reagent according to the manufacturer’s manual (Ambion). One μg of RNA was treated with RNase-Free DNase (Promega). RT reactions were performed with 50 ng/μl oligo-dt (MWG-biotech) and PCR reactions in a total volume of 10 μl containing 2X HotStart Taq master mix (Qiagen), 1 μl of cDNA from the renal cortex RT reaction or 2 μl of cDNA from the DCT2/CNT/iCCD RT reaction, and 1 μM of forward and reverse primers (Table 1). Actin was used as an internal control. The thermal cycling conditions were 1 cycle at 94°C for 15 minutes followed by 30 cycles (renal cortex) or 40 cycles (DCT2/CNT/iCCD) at 94°C for 45 seconds, 61°C for 1 minute, and 72°C for 45 seconds. Reaction products were separated on agarose gels and imaged (BioRad Imager). Reaction products obtained with albumin primers were extracted and sequenced (MWG Biotech).

**Table 1:** Primers used in RT-PCR analyses of albumin mRNA synthesis.

Gene	Sequence 5’-3’	Amplicon
Actin	ACATGGCATTGTTACCAACTGG CGGACTCATCGTACTCCTGCTT	880 bp
eGFP	CCATCCTGATCGAGCTGAATG GACTTGTAGTTGCCGTCATCCTC	286 bp
AQP1	AAGAAGCTCTTCTGGAGGGCTG GCTCACCCGCAACTTCTCAAAC	605 bp
Albumin	CACAAAGATGACAACCCCAGCC GCAGTTTGCTGGAGATAGTCGC	514 bp
Albumin	GCCACCATTTGAAAGGCCAGAG AATCAGCAGCAATGGCAGGCAG	590 bp

### Antibodies

The applied primary antibodies are listed in Table 2. The three anti-albumin antibodies performed equally well in immunoblotting, with lower molecular bands appearing on prolonged exposure for the sheep anti-albumin. Double immunofluorescence labeling shows robust co-localization of the pairwise staining with the rabbit and sheep antibodies as well as with the rabbit and goat antibodies (S1 Fig). Secondary antibodies were: HRP-conjugated goat anti-rabbit or goat anti-mouse IgG (DAKO A/S Denmark), AlexaFluor-488 donkey anti-rabbit, AlexaFluor-488 donkey anti-sheep, AlexaFluor-488 donkey anti-goat, AlexaFluor-488 donkey anti-monkey, AlexaFluor-488 goat-anti rabbit and AlexaFluor-555 donkey anti-rabbit IgG (Invitrogen).

**Table 2:** Primary antibodies used in this study.

Target	Abbreviation	Source	Host
Albumin	Alb	Abcam (19195)	Gt
Albumin	Alb	Abcam (8940–1)	Sh
Albumin	Alb	DAKO (A0001)	Rb
Aldosterone	Aldo	Acris (BP233)	Rb
Aquaporin-2	AQP2	Lofstrand (H7661)	Rb
Anion exchanger 1 (renal)	kAE1	[26]	Rb
Calbindin-D28K	Calbindin	Fitzgerald (10R-C106a)	Mo
Cathepsin D	Cath D	R&D Systems (AF1029)	Gt
Early endosome marker 1	EEA1	BD Biosciences (610457)	Mo
Rab GTPase protein 11	Rab11	BD Biosciences (610656)	Ck
Pendrin	Pendrin	[27]	Rb
V1-ATPase B1 subunit	H^+^-ATPase	[28]	Rb

Gt: goat, Sh: sheep, Rb: rabbit, Mo: mouse, Ck: chicken.

### Statistics

Semi-quantitation based on immunofluorescence histochemistry was tested by ANOVA with Dunnett posttest choosing a significance level of p<0.05.

## Results

### Albumin is found in both proximal tubules and more distal renal tubular segments

**Fig 1:**
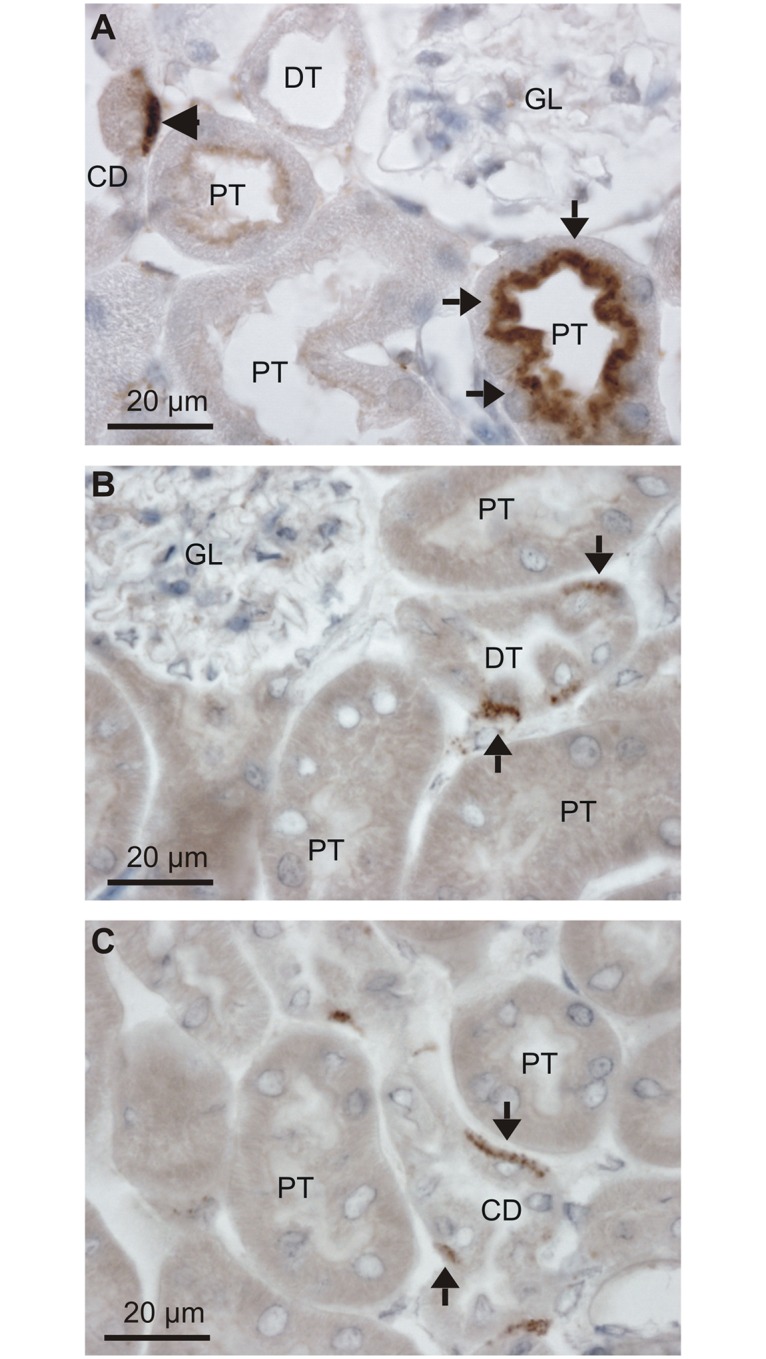
Detection of albumin in the renal cortex. Paraffin wax sections from paraformaldehyde fixed mouse kidneys were immunoperoxidase stained for albumin. A) Immunostaining pattern after labeling with a goat anti-albumin (Table 2). Arrows show the well described endocytosed albumin in proximal tubules, whereas the arrowhead indicates immunoreactivity in a collecing duct. Representative micrographs after labeling with a sheep anti-albumin with arrows indicating a distal tubule (B) and a collecting duct (C). GL is a glomerulus, PT is proximal tubules, DT is distal tubules, and CD is collecting ducts.

Filtered serum albumin is taken up by proximal tubules by receptor mediated endocytosis and immunostaining for albumin produces robust staining in the apical domain of these tubular cells as described by others [16,17]. As illustrated in Fig 1A, close inspection of stained mouse kidneys reveal that albumin is present in both proximal tubules and in the basolateral domain of a subset of tubular cells in more distal tubular segments. This labeling pattern was found using both goat anti-albumin and rabbit anti-albumin antibodies (only the former is shown). Immunolabeling with a sheep anti-albumin produced similar staining of the distal renal tubules and collecting ducts (Fig 1B & 1C, respectively), while the proximal tubular staining was absent. This discrepancy is probably caused by partial breakdown of endocytosed albumin in the proximal tubules, thereby disrupting the antibody binding epitope. Nevertheless, three antibodies immunolocalized albumin to a subset of renal tubular cells positioned in later tubular segments.

### Albumin is located in type A intercalated cells in the distal tubules and cortical collecting ducts

**Fig 2:**
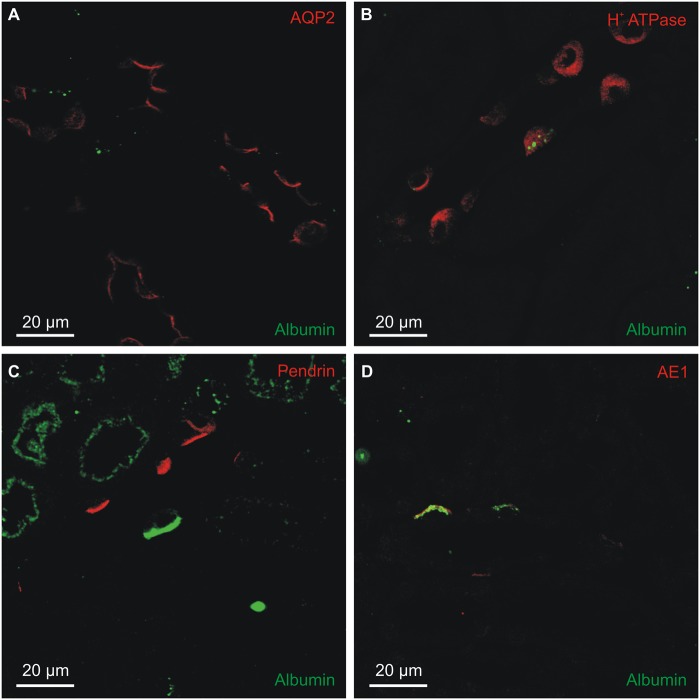
Collecting duct localization of albumin immunoreactivity. Immunofluorescence double-staining was performed for albumin and markers for connecting tubule and collecting duct cells. A) Representative micrograph of sheep anti-albumin labeling (green) and principal cell marker aquaporin-2 staining (AQP2, red). B) Co-staining with sheep anti-albumin (green) and the general intercalated cell marker H^+^-ATPase B1 subunit (red). C) Co-staining with goat anti-albumin (green) and the B-intercalated cell marker pendrin (red). D) Co-staining with sheep anti-albumin (green) and the A-intercalated cell marker anion exchanger 1 (AE1, red).

The identity of the albumin containing cells was determined by double-immunofluorescence staining mouse kidney sections. The intracellular albumin of collecting ducts and connecting tubules did not localize to the AQP2 positive cells principal cells (Fig 2A), and labeling was confined to the H^+^-ATPase positive intercalated cells (Fig 2B). Two main types of intercalated cells have been described: type-A and type-B intercalated cells. Type-B cells express the anion exchanger pendrin in the luminal membrane but did not contain albumin (Fig 2C). The albumin immunoreactivity seemed specific to the type-A cells, as marked by their expression of the renal form of the anion exchanger AE1 in the basolateral membrane (Fig 2D). The intracellular albumin staining appeared in close proximity to the basolateral membrane, i.e. to the AE1 labeling.

### Albumin is confined to membranous intracellular organelles

**Fig 3:**
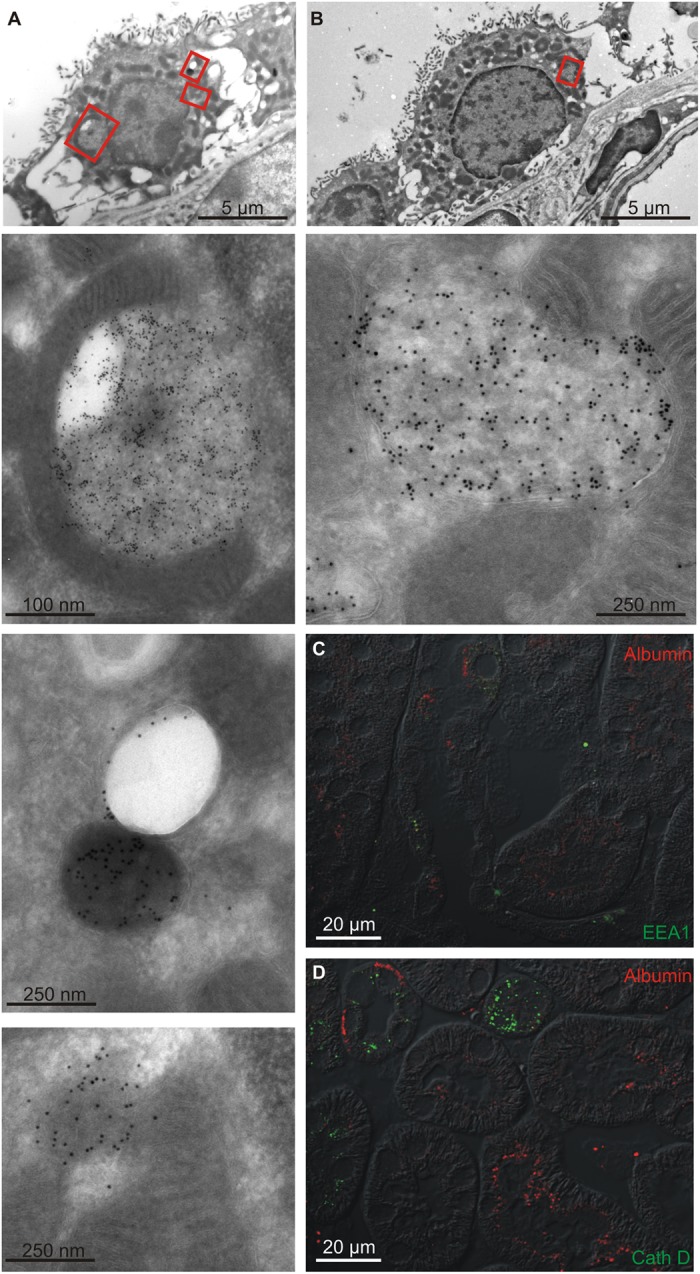
Subcellular localization of albumin in intercalated cells. The subcellular distribution of albumin was assessed by immune-gold electron microscopy and double immunofluorescence histochemistry. A) Top panel: overview micrograph of a mouse renal intercalated cell. The red boxes mark the area magnified in the panels below. Lower panels: high magnification micrograph showing albumin immunoreactivity in spherical structures of varying electron density surrounded by membrane in the basal and mid part of the cell. B) Similar labeling of another intercatated cell. Top panel: overview micrograph of a mouse renal intercalated cell. The red box mark the area magnified in the panel below. Lower panel: high magnification micrograph showing albumin immunoreactivity in a spherical structure surrounded by membrane. C) Double immunolabeling for albumin (red) and early endosome marker EEA1 (green). D) Similar labeling for albumin (red) and the late endosome/lysosome marker Cathepsin D (green). DT = distal tubule/collecting duct, PT = proximal tubule.

Electron microscopy with immune-gold labeling was performed to determine the subcellular localization of albumin in intercalated cells. Fig 3A and 3B show a low magnification micrograph of the intercalated cell in the top panel and details of the same cell at high magnification in the panels below. Albumin is exclusively localized inside the cells in organelles surrounded by a membrane (Fig 3A and 3B). Double immunofluorescence histochemistry was used to assess whether albumin had entered the cell via an endocytotic pathway. EEA1, a marker of early endosomes, only rarely colocalized with albumin (Fig 3C). The marker of late endosomes and lysosomes cathepsin D (not expressed in proximal tubules) did not colocalize with albumin. Not surprisingly, albumin immunoreactivity did not co-localize with apical recycling endosome marker Rab11 (not shown) and to the best of our knowledge there are no reliable antibodies against the basolateral recycling endosomes available for staining paraffin sections. Nevertheless, the albumin in type-A intercalated cells did not seem to arise from uptake of tubular or interstitial albumin by classical endocytosis or to enter the lysosomal system for degradation. We cannot exclude uptake of extracellular albumin by other mechanisms.

### Albumin mRNA is expressed in the renal cortex but not in connecting tubules or collecting ducts

**Fig 4:**
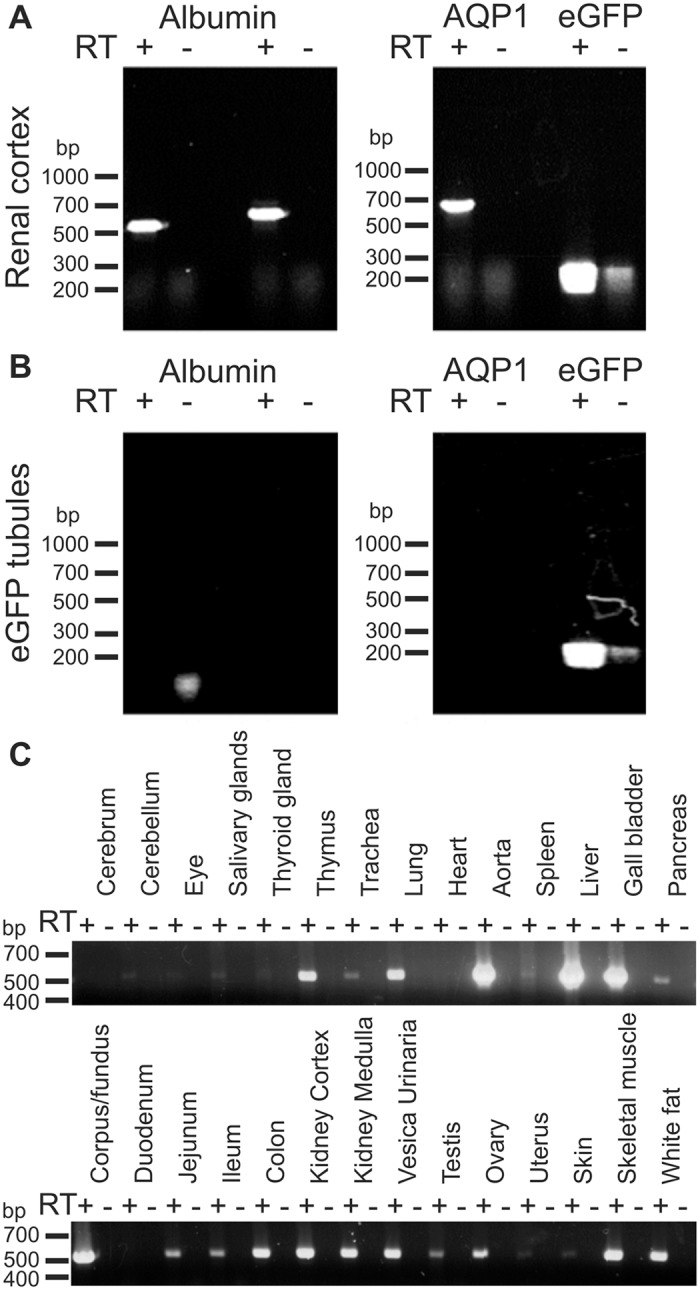
Albumin mRNA expression in kidney cortex and other extrahepatic tissues. Total RNA was extracted from mouse renal cortex and from manually isolated connecting tubules and collecting ducts (A+B). The specific tubules were isolated from mice expressing eGFP in connecting tubules and collecting ducts. A) RT-PCR analysis on renal cortex for albumin, proximal tubule and thin descending limb marker aquaporin-1 (AQP1), and for eGFP on renal cortex. B) Similar analysis performed on isolated eGFP positive tubules. Two sets of albumin primers were applied (Table 2, which includes expected molecular sizes for PCR products). C) Total RNA was extracted from various mouse tissues and subjected to similar RT-PCR analysis for albumin. RT+ and RT- indicate the presence and absence of reverse transcriptase in the transcription reaction.

The pre-urine seems unlikely to be the source of albumin inside the type-A intercalated cells according to the above observations. The alternative sources would be the blood, the interstitium or the renal tubules. RT-PCR on mRNA from whole kidney cortex and isolated connecting tubules and collecting ducts was performed to explore the possibility that albumin could be produced within the renal cortex. The connecting tubules and collecting ducts were isolated based on endogenous fluorescence induced by expression of eGFP under the TRPv5 promoter in these tubules. The renal cortex was positive for mRNA encoding albumin, aquaporin-1 (AQP1, a marker of proximal tubules, descending thin limb and vasa recta), and eGFP (Fig 4A). As expected, the isolated connecting tubules and collecting ducts expressed eGFP, but not AQP1. These tubules were also negative for albumin mRNA (Fig 4B). The analysis was repeated with a separate albumin primer set with identical results and both albumin products from whole renal cortex were verified by nucleotide sequencing. This indicates that cells in the cortex other than the albumin-containing type A intercalated cells have the ability to produce albumin. Nevertheless, the observed renal albumin mRNA expression may be the source of type-A intercalated cell albumin. Several other tissues were also analyzed for albumin mRNA expression to assess the range of extrahepatic albumin mRNA expression. Fig 4C establishes that albumin mRNA is expressed widely in both epithelial and non-epithelial tissues. Thus, hepatocytes may be the only source of circulating albumin, but albumin seems widely expressed probably serving as of yet undefined local functions.

### Renal extratubular albumin immunoreactivity is found in the interstitium

**Fig 5:**
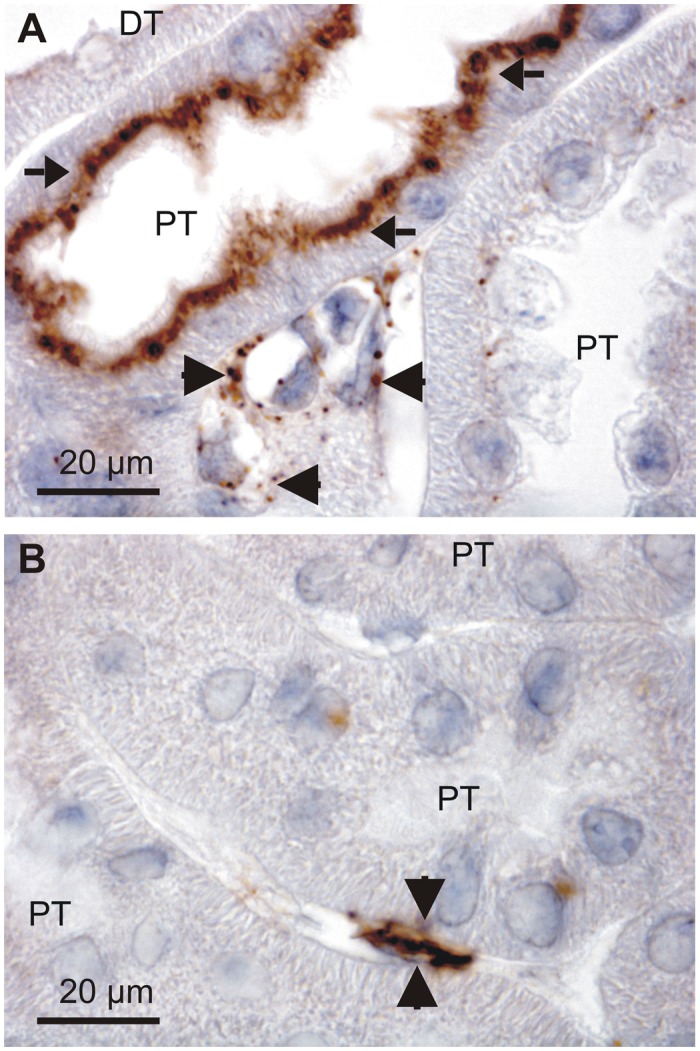
Extratubular albumin expression. Immunoperoxidase staining for albumin in the mouse renal cortex with two anti-albumin antibodies. A) Goat anti-albumin labeling of proximal tubules (arrows), as well as peritubular cells (arrow heads). B) Sheep anti-albumin labeling of the same structures. DT = distal tubule, PT = proximal tubule.

In search of the source of intrarenal albumin, immunperoxidase stained sections were reevaluated. Fig 5A shows both the endocytosed proximal tubular albumin and punctate immunoreactivity in interstitial cells which in some cases surround the microvessels. The same was observed using other anti-albumin antibodies that do not label proximal tubules (Fig 5B). This was also observed in the extra renal tissues positive for mRNA (not shown).

### MDCK cells take up fluorescent albumin from the basolateral side

**Fig 6:**
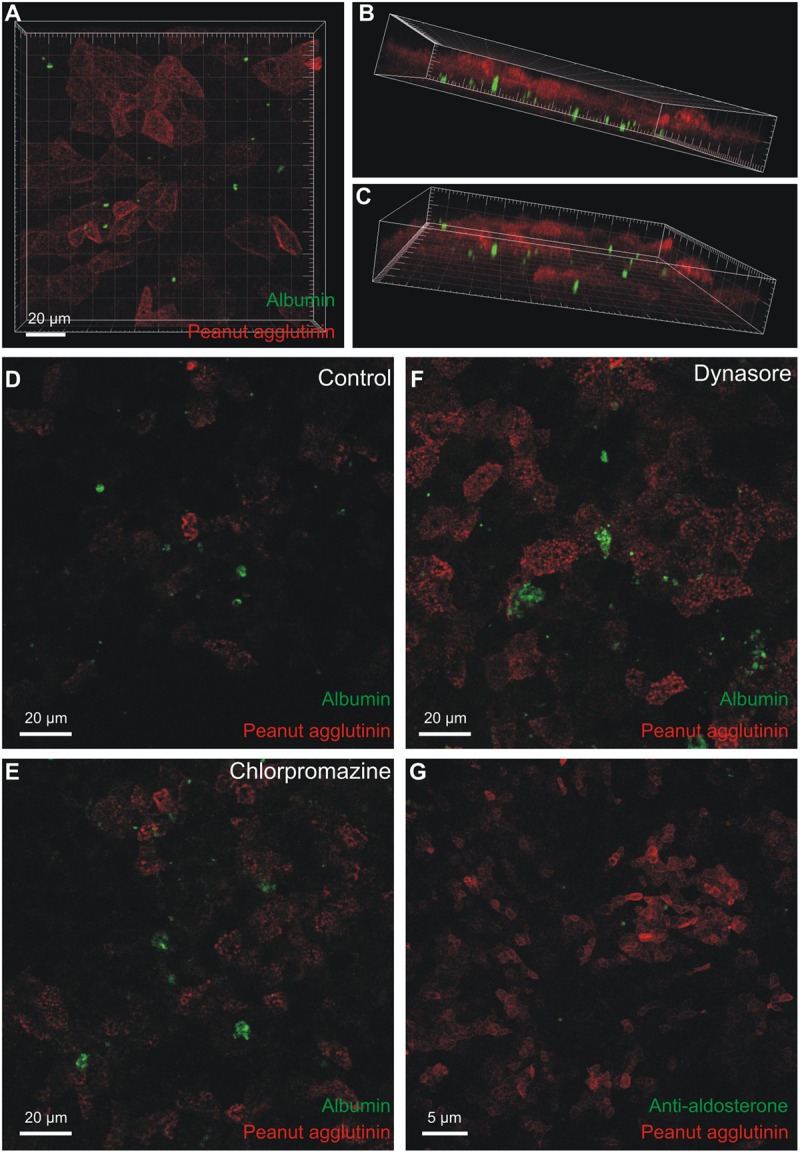
Uptake of fluorescent albumin in MDCK cells. MDCK-I cells were grown on permeable support to confluency. Fluorescence-tagged albumin was added for 24 hours to the lower chamber and the cells were fixed and imaged by confocal microscopy. A) Top view of the stack of images with internalized albumin (green) and rhodamine peanut agglutinin as a luminal surface marker (red). B & C) Oblique views of the same data set showing albumin-fluorescence inside the cells (green). D) Similar uptake of albumin after 24-hours exposure (control for E and F). E) Representative micrograph of albumin uptake after 24 hours in the presence of an inhibitor of clathrin-mediated endocytosis (chlorpromazine), F) a parallel experiment performed in the presence of an inhibitor of dynamin-dependent endocytosis (dynasore). G) 24 hours uptake of aldosterone conjugated albumin, where aldosterone (green) was visualized by immunostaining after fixation and permeabilization. The red signal is peanut agglutinin against the apical membrane.

*In vitro* experiments were conducted to establish whether renal epithelial cells from connecting tubules and collecting ducts are capable of internalizing albumin. The widely used cell line MDCK is derived from these tubular segments and these cells were grown on permeable support. Fluorescein tagged albumin was added to the top bath (representing lumen) or the bottom bath (representing the interstitium) for 8 to 24 hours. Albumin added to the top bath was not taken up by the cells, but seemed to adhere to the external surface in rare cases (not shown, n = 5). By contrast the MDCK cells took up fluorescent albumin when added to the bottom chamber (Fig 6A). The intracellular fluorescein-albumin was observed away from the luminal cell domain marked with peanut agglutinin rhodamine (Fig 6B and 6C) and was more pronounced after 24 hours than after 8 hours of albumin exposure. Interestingly, the albumin seemed to be taken up by cells with low peanut agglutinin binding, indicated that only a subset of MDCK cells took up albumin. Wild type MDCK cells are a mixed population of principal cells, type-A, and type-B intercalated cells. The MDCK c7 clone resembles principal cells, while the MDCK c11 clone resembles type-B intercalated cells [18,19]. None of these cell lines took up basolateral albumin-fluorescein (not shown, n = 2). Thus the observations are consistent with only type-A intercalated cells being capable of internalizing fluorescein-albumin.

As mentioned above, the uptake of albumin into type-A cells seemed unrelated to classical endocytosis and endolysosomal degradation. We studied the effects of chlorpromazine and dynasore on fluorescein-albumin uptake to assess whether the uptake into MDCK cells depended on clathrin-mediated endocytosis or broadly on dynamin-dependent endocytosis, respectively. As exemplified, albumin uptake was neither blocked by chlorpromazine nor dynasore nor indomethacin (Fig 6D–6F, n = 4). The same batch of dynasore was used successfully to inhibit endocytosis in other studies from our department [20]. As for type-A intercalated cells, the intracellular albumin does not accumulate as a result of a classical endocytic pathway. Albumin is a carrier of multiple water insoluble molecules such as free fatty acids and steroid hormones [2]. We speculated that albumin bound to aldosterone would be a physiologically relevant compound to study for renal cell endocytosis. Fig 6G shows intracellular accumulation of aldosterone-albumin complexes after 24 hour basolateral exposure by immnohistochemical labeling for aldosterone in a subset of MDCK cells.

### Albumin abundance in intercalated cells is reduced by aldosterone

**Fig 7:**
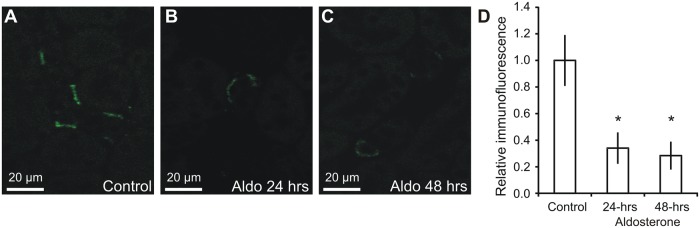
Regulation of albumin by aldosterone. Mice were administered aldosterone for 0, 24 or 48 hours and paraffin sections from fixed kidneys were subjected to immunohistochemical analysis. A) Representative immunostaining for albumin in control mouse kidneys. B) Albumin immunostaining performed in parallel of a mouse kidney after 24 hours aldosterone administration (Aldo). C) Albumin immunostaining of a mouse kidney after 48 hours aldosterone administration. D) Semiquantified albumin abundance from micrographs similar to A-C after correction for background signal, cell area, and normalization to control values (n = 6). * indicates significant changes relative to control.

A previous study from our laboratory indicated that the albumin abundance within connecting tubules and collecting ducts was reduced after short-term aldosterone administration [8]. Mice were subjected to an identical protocol and fixed kidneys were analyzed by immunohistochemistry (Fig 7A–7C). The micrographs are consistent with a reduction in type-A intercalated cell albumin abundance. The albumin immunoreactivity was therefore semiquantified from micrographs from each animal in the three groups. Albumin abundance was decreased by 66% at 24 hours, and by 72% at 48 hours after aldosterone administration compared to controls (Fig 7D, p<0.05, n = 6).

## Discussion

Recent studies have detected albumin protein in isolated cells from late DCT, CNT and CCD by mass spectrometry [8,9] and albumin mRNA was isolated from the renal cortex [21]. However, albumin was not found in the principal cell mpkCCDc14 cell line transcriptome or proteome (http://helixweb.nih.gov/ESBL/Database/index.html), or in the rat IMCD transcriptome [22]. In the present study, we revisited the putative albumin content in late DCT, CNT and CCD and confirm evidence for intracellular albumin in cells from the renal tubular segments other than proximal tubules. Albumin immunoreactivity was confined to type-A intercalated cells in the late DCT, CNT and CCD, not previously reported to contain albumin.

Until recently, renal albumin was only described within the blood vessels, the ultrafiltrate and to endocytotic vesicles of proximal tubules [4,6]. The albumin in type-A cells we describe may arise from either endocytosis or *de novo* synthesis in these cells and the first source of tubular albumin may be filtered serum albumin. However, there are three reasons to doubt that the albumin content in type-A cells results from reabsorption from the preurine. Firstly, serum albumin filtered in the glomeruli is efficiently reabsorbed by the proximal tubules and the content of albumin in the preurine reaching the distal tubular system is normally negligible [23]. Secondly, the distal tubular segments are not known to express receptors for macromolecule endocytosis such as megalin and cubilin [16,17]. Thirdly, albumin was predominantly found in the basal domain of the cell and not in the luminal domain as in the proximal tubules.

As noted above, albumin was found in the basolateral domain of the cell inside a membrane delimited organelle. This would be expected both if albumin was taken up by the cell and if the cell had produced the protein *de novo* and stored it in exocytotic vesicles. However, the protein was not associated with the normal endo-lysosomal system in the late DCT, CNT and CCD. Therefore, we hypothesized that albumin may be produced by these cells instead of internalizing the protein from the exterior environment. However, isolated late DCT, CNT and CCD segments did not express albumin mRNA in repeated analysis. This is consistent with the cessation of tubular albumin mRNA expression shortly after birth in mouse kidney as analyzed by *in situ* hybridization [24,25]. In embryology, thus, extrahepatic expression of albumin is well established, but renal albumin mRNA expression in the adult kidney is only associated with acute kidney injury [21]. We find albumin mRNA expression in the normal adult kidney. The mRNA does not seem to stem from the tubular system, but a non-tubular cell type in the renal cortex. We failed to identify these cells by *in situ* hybridization and by expressing eGFP in the kidneys under control of the albumin promoter (albumin promoter driven Cre expressing mice crossed with a DsRed-EGFP reporter mouse: B6.Cg-Tg(CAG-DsRed,-EGFP)5Gae/J). However, we observe albumin immunoreactivity in interstitial cells in the kidneys from adult mice and speculate that these cells may be the source of type-A intercalated cell albumin. Interstitial cells are common to many tissues, and therefore the expression of albumin mRNA in a variety of epithelial and non-epithelial tissues was supportive for this hypothesis.

The source of albumin in type-A cells may, thus, be the renal cortical interstitium and the protein would be taken up across the basolateral membrane. We shifted to an *in vitro* model, to test the putative capability of cells in the CNT and CCD to internalize albumin from the basal side directly. Indeed, MDCK cell cultures took up albumin from the basolateral side only. Native albumin, fluorescein-albumin, as well as albumin bound to aldosterone were taken up in by the cells. However, again the mechanism of internalization may not be classical clathrin mediated endocytosis or other dynamin dependent pathways, as neither chlorpromazine nor dynasore blocked the uptake. The lack of internalization in pure populations of principal cells and type-B intercalated cells indicate that albumin was taken up by type-A cells also in the mixed wildtype MCDK cultures.

The rationale for interstitial cells to potentially produce and secrete albumin and for type-A intercalated cells to internalize the protein remains elusive. Lipophilic substances carried by serum albumin in the blood are believed diffuse in unbound form across the blood vessels and through the interstitium to reach the target cells. Here, these substances would bind to a receptor for endocytosis or integrate into the lipid membrane directly. In future studies, it would be most interesting to assess whether lipophilic substances such as free fatty acids and steroid hormones diffuse from the blood vessels to the epithelial cells through the hydrophilic interstitium bound to locally produced albumin.

We previously demonstrated aldosterone-regulated albumin abundance in mouse late DCT, CNT and CCD. As described above, the current study verifies the presence of albumin in these tubules and specifies the cell type that contains the protein. In addition, mice displayed significantly decreased albumin abundance in the type-A intercalated cells after 24 hours of aldosterone administration. One can only speculate why albumin abundance would be negatively regulated by aldosterone. The specific uptake of albumin into the type-A intercalated cells may indicate that these cells are either the main target for substances carried by interstitial albumin or responsible for the degradation of interstitial albumin. The lack of co-localization of albumin and the endo-lysosomal degradation system seems to contradict the latter possibility. The former possibility is supported by the fact that late DCT, CNT and CD comprise the aldosterone sensitive renal tubules, but is challenged by the cellular aldosterone sensitivity, which is not restricted to the intercalated cells. Despite our finding that CNT/CCD cell cultures (MDCK) are capable of taking up aldosterone bound to albumin, we do not know which substances are taken up by type-A cells together with albumin *in vivo*. At this time, we can only claim that aldosterone administration reduces albumin abundance in type-A cells by decreasing local albumin production, increasing the rate of albumin degradation or reducing the internalization into type-A cells.

In conclusion, we present evidence of intracellular albumin in type-A intercalated cells in the mouse kidney cortex and that CNT/CCD cells (MDCK) take up unconjugated albumin, albumin-fluorescein, as well as aldosterone-albumin complexes from the basolateral side. We speculate that albumin may be produced in the interstitial cells and serve as a carrier for lipophilic substances from the blood to the kidney cells. We show that the extrahepatic albumin expression is a general phenomenon and that the albumin content in type-A intercalated cells is dynamic, i.e. sensitive to hormonal challenges. Further studies should focus on determining which substances are bound to albumin in type-A cells and in the renal interstitium to promote exploration of the physiological significance of this possibly extrahepatic albumin synthesis.

## Supporting Information

S1 Fig Three anti-albumin antibodies were applied in this study. A) Proteins from mouse renal cortex homogenate were separated by SDS-PAGE and immunoblotted with rabbit anti-albumin (DAKO), goat anti-albumin (Abcam), and sheep anti-albumin (Abcam). Overexposure of the goat anti-albumin blot did not reveal additional immunoreactive bands than the expected band for albumin. B) Overexposure of blots with the rabbit anti-albumin and sheep anti-albumin antibodies revealed weak bands only for the sheep anti-albumin antibody. Normal exposure for goat anti-albumin is also shown. C) Double immunofluorescence labeling with rabbit and goat anti-albumin antibodies revealed a high degree of colocalization of the staining in the two color channels. D) Double immunofluorescence labeling with rabbit and sheep anti-albumin antibodies also revealed a high degree of colocalization of the staining in the two color channels.
(TIF)

## References

[1] Weisberg HF. Osmotic pressure of the serum proteins. Ann Clin Lab Sci. 1978;8:155–164. 345945

[2] Kragh-Hansen U. Molecular aspects of ligand binding to serum albumin. Pharmacol Rev. 1981;33:17–53. 7027277

[3] Brenner BM, Baylis C, Deen WM. Transport of molecules across renal glomerular capillaries. Physiol Rev. 1976;56:502–534. 778868 10.1152/physrev.1976.56.3.502

[4] Maunsbach AB. Albumin absorption by renal proximal tubule cells. Nature. 1966;212:546–547. 5970207 10.1038/212546a0

[5] Schwegler JS, Heppelmann B, Mildenberger S, Silbernagl S. Receptor-mediated endocytosis of albumin in cultured opossum kidney cells: a model for proximal tubular protein reabsorption. Pflugers Arch. 1991;418:383–392. 1652125 10.1007/BF00550876

[6] Christensen EI, Nielsen S. Structural and functional features of protein handling in the kidney proximal tubule. Semin Nephrol. 1991;11:414–439. 1947495

[7] Clapp WL, Park CH, Madsen KM, Tisher CC. Axial heterogeneity in the handling of albumin by the rabbit proximal tubule. Lab Invest. 1988;58:549–558. 3367637

[8] Jensen TB, Pisitkun T, Hoffert JD, Jensen UB, Fenton RA, Praetorius HA, et al Assessment of the effect of 24-hour aldosterone administration on protein abundance in fluorescence-sorted mouse distal renal tubules by mass spectrometry. Nephron Physiol. 2012;121:9–15. 10.1159/000346832 PMC3648998 23428628

[9] Da Silva N, Pisitkun T, Belleannee C, Miller LR, Nelson R, Knepper MA, et al Proteomic analysis of V-ATPase-rich cells harvested from the kidney and epididymis by fluorescence-activated cell sorting. Am J Physiol Cell Physiol. 2010;298:C1326–1342. 10.1152/ajpcell.00552.2009 20181927 PMC2889637

[10] Brown D, Bouley R, Paunescu TG, Breton S, Lu HA. New insights into the dynamic regulation of water and acid-base balance by renal epithelial cells. Am J Physiol Cell Physiol. 2012;302:C1421–1433. 10.1152/ajpcell.00085.2012 22460710 PMC3362000

[11] Biner HL, Arpin-Bott MP, Loffing J, Wang X, Knepper M, Hebert SC, et al Human cortical distal nephron: distribution of electrolyte and water transport pathways. J Am Soc Nephrol. 2002;13:836–847. 11912242 10.1681/ASN.V134836

[12] van der Lubbe N, Lim CH, Meima ME, van Veghel R, Rosenbaek LL, Mutig K, et al Aldosterone does not require angiotensin II to activate NCC through a WNK4-SPAK-dependent pathway. Pflugers Arch. 2012;463:853–863. 10.1007/s00424-012-1104-0 22549242 PMC3350624

[13] Masilamani S, Kim GH, Mitchell C, Wade JB, Knepper MA. Aldosterone-mediated regulation of ENaC alpha, beta, and gamma subunit proteins in rat kidney. J Clin Invest. 1999;104:R19–23. 10510339 10.1172/JCI7840 PMC408561

[14] Winter C, Kampik NB, Vedovelli L, Rothenberger F, Paunescu TG, Stehberger PA, et al Aldosterone stimulates vacuolar H(+)-ATPase activity in renal acid-secretory intercalated cells mainly via a protein kinase C-dependent pathway. Am J Physiol Cell Physiol. 2011;301:C1251–1261. 10.1152/ajpcell.00076.2011 21832245 PMC3213922

[15] Hofmeister MV, Fenton RA, Praetorius J. Fluorescence isolation of mouse late distal convoluted tubules and connecting tubules: effects of vasopressin and vitamin D3 on Ca2+ signaling. Am J Physiol Renal Physiol. 2009;296:F194–203. 10.1152/ajprenal.90495.2008 18987111

[16] Birn H, Fyfe JC, Jacobsen C, Mounier F, Verroust PJ, Orskov H, et al Cubilin is an albumin binding protein important for renal tubular albumin reabsorption. J Clin Invest. 2000;105:1353–1361. 10811843 10.1172/JCI8862 PMC315466

[17] Cui S, Verroust PJ, Moestrup SK, Christensen EI. Megalin/gp330 mediates uptake of albumin in renal proximal tubule. Am J Physiol. 1996;271:F900–907. 8898021 10.1152/ajprenal.1996.271.4.F900

[18] Gekle M, Wunsch S, Oberleithner H, Silbernagl S. Characterization of two MDCK-cell subtypes as a model system to study principal cell and intercalated cell properties. Pflugers Arch. 1994;428:157–162. 7971172 10.1007/BF00374853

[19] Wunsch S, Gekle M, Kersting U, Schuricht B, Oberleithner H. Phenotypically and karyotypically distinct Madin-Darby canine kidney cell clones respond differently to alkaline stress. J Cell Physiol. 1995;164:164–171. 7790388 10.1002/jcp.1041640121

[20] Rosenbaek LL, Kortenoeven ML, Aroankins TS, Fenton RA. Phosphorylation decreases ubiquitylation of the thiazide-sensitive cotransporter NCC and subsequent clathrin-mediated endocytosis. J Biol Chem. 2014;289:13347–13361. 10.1074/jbc.M113.543710 24668812 PMC4036343

[21] Ware LB, Johnson AC, Zager RA. Renal cortical albumin gene induction and urinary albumin excretion in response to acute kidney injury. Am J Physiol Renal Physiol. 2011;300:F628–638. 10.1152/ajprenal.00654.2010 21147844 PMC3064135

[22] Uawithya P, Pisitkun T, Ruttenberg BE, Knepper MA. Transcriptional profiling of native inner medullary collecting duct cells from rat kidney. Physiol Genomics. 2008;32:229–253. 17956998 10.1152/physiolgenomics.00201.2007 PMC2276652

[23] Boron W, Boulpaep EL. Medical Physiology: Saunders Elsevier 2009.

[24] Poliard A, Feldmann G, Bernuau D. Alpha fetoprotein and albumin gene transcripts are detected in distinct cell populations of the brain and kidney of the developing rat. Differentiation. 1988;39:59–65. 2469611 10.1111/j.1432-0436.1988.tb00081.x

[25] Moorman AF, De Boer PA, Evans D, Charles R, Lamers WH. Expression patterns of mRNAs for alpha-fetoprotein and albumin in the developing rat: the ontogenesis of hepatocyte heterogeneity. Histochem J. 1990;22:653–660. 1706693 10.1007/BF01047449

[26] Christensen BM, Kim YH, Kwon TH, Nielsen S. Lithium treatment induces a marked proliferation of primarily principal cells in rat kidney inner medullary collecting duct. Am J Physiol Renal Physiol. 2006;291:F39–48. 16434572 10.1152/ajprenal.00383.2005

[27] Kim YH, Kwon TH, Frische S, Kim J, Tisher CC, Madsen KM, et al Immunocytochemical localization of pendrin in intercalated cell subtypes in rat and mouse kidney. Am J Physiol Renal Physiol. 2002;283:F744–754. 12217866 10.1152/ajprenal.00037.2002

[28] Nelson RD, Guo XL, Masood K, Brown D, Kalkbrenner M, Gluck S. Selectively amplified expression of an isoform of the vacuolar H(+)-ATPase 56-kilodalton subunit in renal intercalated cells. Proc Natl Acad Sci U S A. 1992;89:3541–3545. 1373501 10.1073/pnas.89.8.3541 PMC48904

