# Supplementary figures and images for: Renal Type A Intercalated Cells Contain Albumin in Organelles with Aldosterone-Regulated Abundance
Source: PLoS One. 2015 Apr 13;10(4):e0124902. doi: 10.1371/journal.pone.0124902 (PMC4395387; doi:10.1371/journal.pone.0124902)

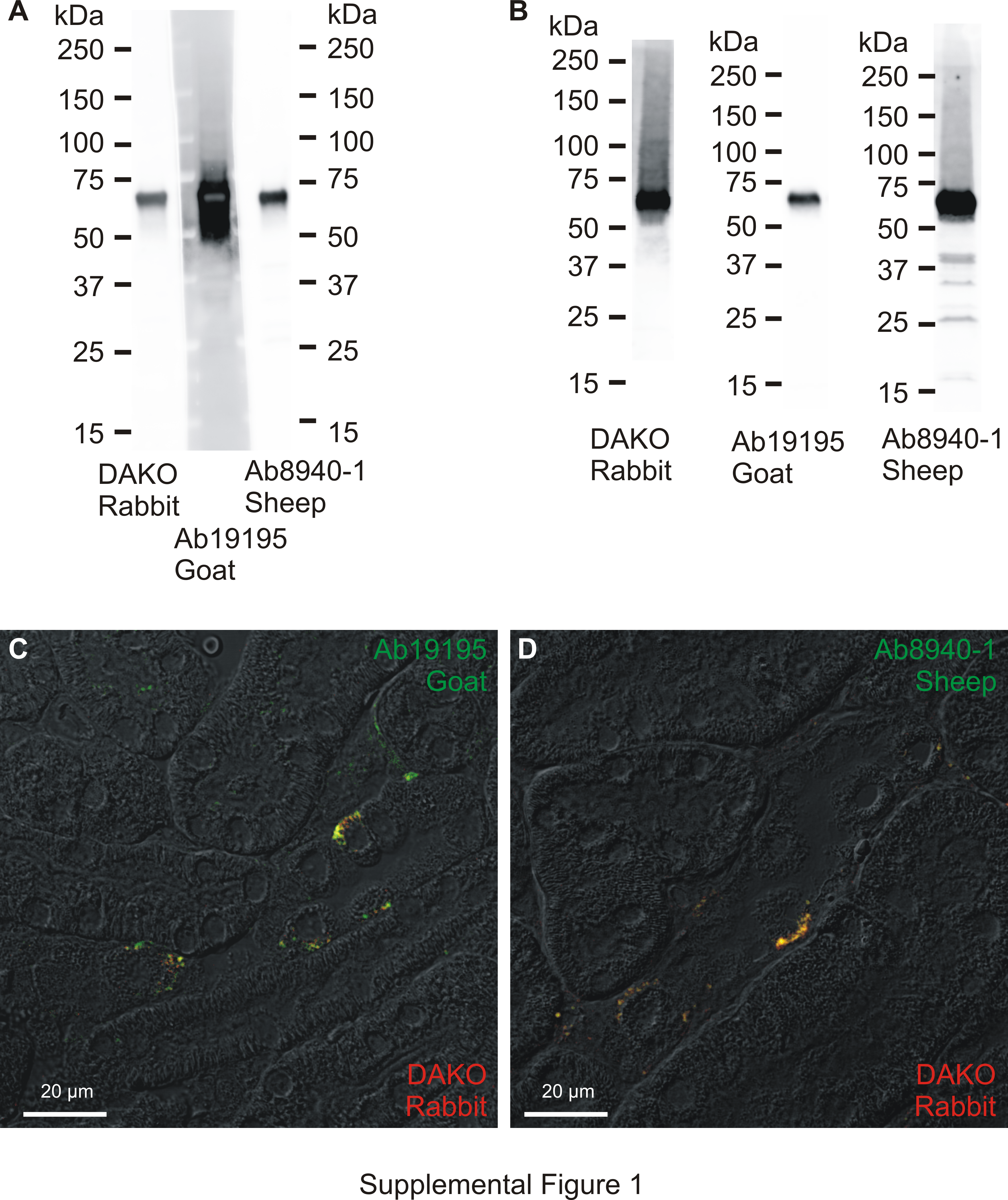

Supplement: S1 Fig — A) Proteins from mouse renal cortex homogenate were separated by SDS-PAGE and immunoblotted with rabbit anti-albumin (DAKO), goat anti-albumin (Abcam), and sheep anti-albumin (Abcam). Overexposure of the goat anti-albumin blot did not reveal additional immunoreactive bands than the expected band for albumin. B) Overexposure of blots with the rabbit anti-albumin and sheep anti-albumin antibodies revealed weak bands only for the sheep anti-albumin antibody. Normal exposure for goat anti-albumin is also shown. C) Double immunofluorescence labeling with rabbit and goat anti-albumin antibodies revealed a high degree of colocalization of the staining in the two color channels. D) Double immunofluorescence labeling with rabbit and sheep anti-albumin antibodies also revealed a high degree of colocalization of the staining in the two color channels. (TIF) [file pone.0124902.s001.tif]
